# Abiotic stress-induced secondary metabolite production in Brassica: opportunities and challenges

**DOI:** 10.3389/fpls.2023.1323085

**Published:** 2024-01-04

**Authors:** Muthusamy Muthusamy, Soo In Lee

**Affiliations:** Department of Agricultural Biotechnology, National Institute of Agricultural Sciences (NAS), Rural Development Administration, Jeonju, Republic of Korea

**Keywords:** Brassica, abiotic stress, glucosinolates, flavonoids, carotenoids, phenolic acids, alkaloids

## Abstract

Over the decades, extensive research efforts have been undertaken to understand how secondary plant metabolites are affected by genetic, environmental, and agronomic factors. Understanding the genetic basis of stress-response metabolite biosynthesis is crucial for sustainable agriculture production amidst frequent occurrence of climatic anomalies. Although it is known that environmental factors influence phytochemical profiles and their content, studies of plant compounds in relation to stress mitigation are only emerging and largely hindered by phytochemical diversities and technical shortcomings in measurement techniques. Despite these challenges, considerable success has been achieved in profiling of secondary metabolites such as glucosinolates, flavonoids, carotenoids, phenolic acids and alkaloids. In this study, we aimed to understand the roles of glucosinolates, flavonoids, carotenoids, phenolic acids and alkaloids in relation to their abiotic stress response, with a focus on the developing of stress-resilient crops. The focal genus is the Brassica since it (i) possesses variety of specialized phytochemicals that are important for its plant defense against major abiotic stresses, and (ii) hosts many economically important crops that are sensitive to adverse growth conditions. We summarize that augmented levels of specialized metabolites in Brassica primarily function as stress mitigators against oxidative stress, which is a secondary stressor in many abiotic stresses. Furthermore, it is clear that functional characterization of stress-response metabolites or their genetic pathways describing biosynthesis is essential for developing stress-resilient Brassica crops.

## 1 Introduction

**Figure 1:**
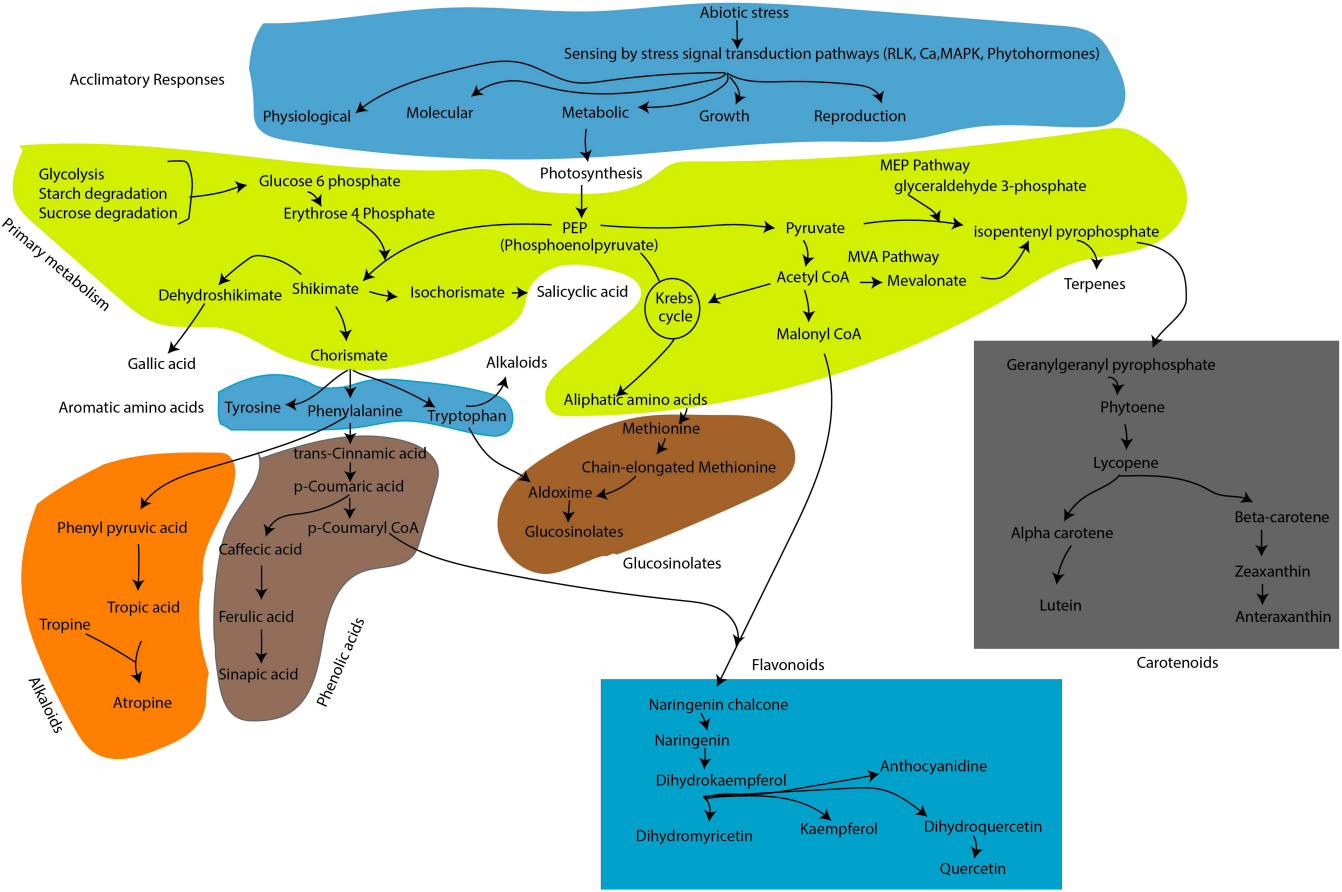
Schematic representation of metabolic pathways leading to secondary metabolite production in response to plant stress signals. For convenience of the readers, cascades of enzymatic reaction or other regulatory elements and intermediate biosynthesis were excluded in this figures. In figure, the biosynthesis pathways of flavonoid, carotenoid, phenolic acid, glucosinolates and alkaloids were illustrated among the several known secondary metabolites in plants.

Climate change will intensify the adverse effects of both biotic and abiotic stress conditions, thus threatening agricultural sustainability and ultimately leading to economic losses (Lozano-Elena et al., 2022). Furthermore, it is eminent that future predictions and forecasts on climatic conditions indicate the possibility of an increase in the frequency and severity of major abiotic stress constraints, including drought, salinity, cold, and low/high temperatures. The plant stress response pathway is highly intricate and regulated at multiple levels through integrated and coordinated signals perceived by sensors to adjust or adapt to adverse conditions. Therefore, it is important to identify suitable screening indices and quantifiable traits to facilitate the production of stress-resilient crops. For this purpose, the top priorities will include gaining insights into the role of multiple players at physiological, biochemical, and molecular levels in plant stress response mechanisms. To date, there have been several comprehensive analyses of the molecular biology of plant stress adaptive mechanisms, and the outcomes have been useful in significantly improving key agronomic traits such as plant growth and yield performance in stressed crops. Nevertheless, it is essential to implement innovative and alternative approaches considering the bottlenecks in our understanding the plants stress responses, given their polygenic nature and the occurrence of more prevalent episodes of climate abnormalities. It is known that plants continuously evolve to mitigate the stressors by developing constitutive, active, inducible, and tightly regulated immune and defense systems. In terms of metabolic adjustments to stress signals, plant cells coordinate their metabolic pathways to produce anti-stress agents focused on maintaining homeostasis inside cells. A generalized metabolic pathways leading to secondary metabolite biosynthesis/production was described in **Figure 1**.

Among the plant stress mitigation strategies, accumulation of secondary metabolites with selective stress-protective effects against growth limiting environmental constraints has been fairly studied and it continues to evolve with the assistance of technical advancements in specific and targeted metabolite profiling and quantification approaches. The literature shows that information on stress-response metabolite profile is rapidly expanding in recent times, indicating their potential role in plant stress metabolic adaptation (Chan et al., 2013). Synchronization with environmental growth constraints, factors such as diurnal or circadian oscillations also exert influence over the production of defense chemicals in plants (Doghri et al., 2022). This indicates that multiple factors modulate secondary metabolite production, which makes our tasks difficult in identification of precise metabolites for a particular stress conditions. This may be one of the reasons why the success story of producing climate-resilient crops is still elusive. Metabolomics approaches targeting qualitative and quantitative profiling of phytochemicals in plants involved in stress responses could provide valuable information about the metabolic adaptation of plants under adverse conditions (Shaw et al., 2022) since the metabolic profile has a direct relationship with the phenotype (Viana et al., 2022). Additionally, genetic pathways describing the metabolite biosynthesis and regulatory elements that control metabolite accumulation/reduction could offer the necessary information for genetic manipulation strategies aiming at developing stress-smart crops.

In general, plant metabolism can be influenced by several factors, including climatic factors (such as season, light, water, temperature, CO_2_ levels, air and soil pollutants), biotic factors (including plant species, genotype, pests, pathogens, and competitive habitats/weeds), and agronomic factors (such as soil type, fertilizer type, pesticides, cultural practices, and habitat manipulation) (Björkman et al., 2011). It is widely believed that over 50% of the estimated secondary metabolites (over 200,000) in plants are thought to be influenced by stress conditions, and some of these compounds could serve various defense roles in plants, including acting as antioxidants, antifungal agents, antibacterial agents, ant herbivore deterrents, and stress-inducible phytohormones, among others (Kessler and Kalske, 2018). The plant defense compounds are diverse in nature and comprises of several known metabolites such as terpenoids, alkaloids, phenolics, steroids, flavonoids, tannins, and possibly many other unidentified metabolites (Pant et al., 2021). In most cases, stress response production of secondary metabolites such as glucosinolates, carotenoids, flavonoids, phenolic acids, and alkaloids enhance plant antioxidant efficiencies through mitigating the oxidative stress, which is a secondary stressor of major abiotic stresses in plants, such as drought, salinity, cold and high temperature, among others. These plant defense associated secondary metabolites generally show increased levels after encountering both biotic and abiotic stresses. It is worth noting that these phytochemicals and their degradation products, possesses a wide range of biological properties, including antioxidant, anticancer, antimicrobial, anti-inflammatory, antidiabetic, and neuroprotective activities in the human diet (Ayadi et al., 2022). Consequently, abiotic stress factors that influence the quantity and quality of metabolite content may impact the nutritional benefits for both animal and human health. Therefore, controlling the optimal growth factors in crop plants is essential to harness the specific metabolite accumulation and/or maintaining desired metabolite profiles in edible parts of the plants and more importantly to maintain the intrinsic quality, including color, aroma, taste, and beneficial health properties of host plants. For instance, excess accumulation of certain glucosinolates (GSLs) acts as anti-nutritional factors in Brassica plants (Miao et al., 2021). Nonetheless, these stress-induced specialized compounds are valuable in mitigating adverse growth conditions through multiple mechanisms (Björkman et al., 2011). Also, previous studies analyzing the stress-responsive metabolic adaptation potential of glucosinolates, sterols, terpenes, and flavonoids proved their biological significance in plant abiotic stress responses (Nephali et al., 2020). Considering the biological significance of several known and a larger fraction of unknown defense chemicals, their diversities and multifactor-driven production mechanisms it is clear that our current understanding of the stress responsive metabolic adaptation of pants is far from complete. However, it is encouraging that modern technical advances in farming practices and analytical chemistry has enabled the identification of metabolites that accumulate under particular physiological conditions (Ljubej et al., 2021c), thereby enhancing our knowledge of their biological significance.

Our area of interest is the Brassica genus, commonly grown in the Mediterranean area, and their production is greatly affected by unfavorable environmental conditions. Brassica crops are widely cultivated due to its nutritional and economic significance within the Brassicaceae family, commonly referred to as crucifers. Considering this significance and the frequent occurrence of environmental growth constraints, it is crucial for the research community to develop strategies for dissecting the metabolic adaptation mechanism under stress conditions in Brassica vegetables and oils (Wu et al., 2021). The principal vegetable species belonging to the Brassica genus include *Brassica oleracea* (e.g., broccoli, cabbage, cauliflower, kale, Brussels sprouts, etc.), *Brassica rapa* (e.g., turnip, Chinese cabbage, and pak choi), *Brassica napus* (e.g., rapeseed and leaf rape), *Brassica juncea*, and *Brassica carinata* (mustards). It includes 39 species, many of which are important agricultural and horticultural crops with different edible organs such as inflorescences (e.g., broccoli and cauliflower), leaves (e.g., kale and pak choi), heads (e.g., white and red cabbage), as well as roots and bulbs (e.g., radish and turnip). They serve as vegetables, oilseed crops, and forage species (Neugart et al., 2018; Poveda et al., 2020; Ayadi et al., 2022). The Brassica genus consists of three diploid species (*Brassica rapa* L. (e.g., turnip, Chinese cabbage, and pak choi), *Brassica oleracea* L. (e.g., broccoli, cabbage, cauliflower, kale, Brussels sprouts, and kohlrabi), and *Brassica nigra* L. and three amphidiploids such as *Brassica napus* L (e.g., rapeseed or canola and leaf rape), *Brassica carinata* (mustards), and *Brassica juncea* (L.) (mustards) (He et al., 2021). Rapeseed has remained an important crop in the production of edible and non-edible oils for thousands of years (Wang et al., 2022).

With this background information, in this study, we conducted comprehensive analyses of abiotic stress-induced secondary metabolite production in Brassica, namely, glucosinolates, carotenoids, flavonoids, phenolic acids, and alkaloids. This study could facilitate an understanding of their stress-specific and non-specific roles in metabolic adaptation to adverse growth conditions. Altogether, this study presents the starting point for an understanding of the stress response metabolic adaptation in Brassica, which expected to provide a potential metabolite or its biosynthesis candidate gene for improved stress management in Brassica crops. It also offers convenient and alternative approaches for managing short-term abiotic stress conditions through exogenous application of potential stress-mitigating metabolites in crop plants as shown previously (Hasanuzzaman et al., 2014; Linić et al., 2021; Hong et al., 2023). Additionally, it can be useful in harnessing the desired metabolic composition for the benefit of consumers through effective and controlled stress management strategies.

## 2 Glucosinolates

Glucosinolates (GSLs) are a class of sulfur- and nitrogen-containing secondary metabolites derived from amino acids, mainly found in Brassica crops, such as broccoli, cabbage, and oilseed rape (Tang et al., 2023b). The bioactive GSL metabolites confer benefits to plant defense, human health, and the unique flavor of some Brassica crops (Miao et al., 2021). They play multifunctional roles in plants, including stress response, growth, and development. Being specialized metabolites of Brassica, the diverse functions of GSLs and their degradation products under different physiological conditions have been fairly studied (Shaw et al., 2022). Studies have pointed out that GSL content and profiles can differently impact plant fitness under stressful conditions (Klein and Papenbrock, 2008; Muthusamy et al., 2021). On the basis of structure, GSLs can be classified into aliphatic, indolic and aromatic compounds, constituting a total of 200 types of GSLs with various substituents (Martínez-Ballesta et al., 2013). The role of GSLs in different biotic stress responses and their importance in offering stress tolerance have been well-documented until recently(Martínez-Ballesta et al., 2013; Liu et al., 2021; Abuyusuf et al., 2023). However, the responses of GSL metabolism to abiotic stress are relatively understudied.

In an attempt to identify the molecular basis of the low-temperature stress tolerance in *Brassica oleracea* var. *acephala*, it was revealed that chilling increase the total amount of aliphatic glucosinolates, while freezing increase the total amount of indolic glucosinolates, including glucobrassicin (Ljubej et al., 2021b). In another study, BrPP5 overexpression-mediated thermotolerance in *Brassica rapa* L. ssp. *pekinensis* demonstrated that stress tolerance in transgenic lines positively correlated with enhanced accumulation of total GSL content compared to that of wild-type lines (Muthusamy et al., 2021), possibly indicating that GSL content is crucial in determining high-temperature stress tolerance. Individual profiling showed that the concentration of glucobrassicin, 4-methoxyglucobrassicin, neoglucobrassicin and gluconasturtiin were elevated in thermotolerant lines. In case of short-term high-temperature stress (40°C for 8 h) on pakchoi seedlings increased both aliphatic and indolic glucosinolates, including 4-methoxy-glucobrassicin (Rao et al., 2021). In another study investigating the specialized volatiles responding to chronic and short-term heat stress, it was demonstrated that glucosinolate volatiles constitute a major part of emission blend in heat-stressed *B. nigra* plants, especially under chronic stress (Kask et al., 2016). These results suggest that GSL volatiles could constitute specialized volatile defense pathways against heat stress in Brassica crops. Similarly, drought stress could also increase the total GSL content in *Brassica rapa* L. ssp. *pekinensis*. Detailed analysis revealed that indolic GSLs were higher than those in controls (Shawon et al., 2020). Studies also revealed that drought-induced accumulation of glucosinolates in *Brassica rapa* L. ssp. *pekinensis* leaves directly or indirectly control stomatal closure to prevent water loss under drought stress conditions (Eom et al., 2018). This study showed that glucobrassicanapin and 4-methoxyglucobrassicin were abundantly found in drought-stressed plants compared to controls. In particular, there was approximately a six-fold increase in levels of gluconapin and 4-hydroxyglucobrassicin under drought conditions. In contrast, drought exposure had no impact on GSL production, with the exception of glucoraphanin in Pak choi (*Brassica rapa* subsp. *chinensis*) (Park et al., 2021). Furthermore, it was shown that prolonged drought exposure had a positive correlation with glucoraphanin accumulation in Pak choi plants. GSLs and ITCs (GSL derivatives) under drought stress promote stomatal closure by stimulating the production of reactive oxygen species (ROS) (Salehin et al., 2019). Drought stress in curly kale (*Brassica oleracea* L. convar. *Acephala* (DC) var. *sabellica*) increased the expression of genes associated with glucosinolates, especially aliphatic alkenyl glucosinolates and genes associated with glucosinolate breakdown (Podda et al., 2019). It was also shown that drought stress in *Brassica oleracea* L. crops influences both qualitative and quantitative changes in GSL content and suggests a possible link between water stress resistance and the accumulation of NGBS (Ben Ammar et al., 2023). They also revealed that the highest increase in the glucosinolate amount was detected in broccoli roots, followed by cauliflower leaves under water stress conditions. As of now, the impact of GSL accumulation on drought stress responses of those cultivars is not clear. The stress-induced secondary metabolite accumulation also depends on the developmental stages of plants. For instance, reduced UVB and UV exposure in Pak choi sprouts elevated the concentrations of glucosinolates and their hydrolysis products, while similar growth conditions in mature 15- and 30-leaf plants improved the concentrations of flavonoids, hydroxycinnamic acids, carotenoids, and chlorophylls (Heinze et al., 2018). Apart from that Pak choi plants under long-day growth conditions were shown to enhance GSL production, especially gluconapin and neoglucobrassicin indicating GSLs also could respond according to light quantities.

**Figure 2:**
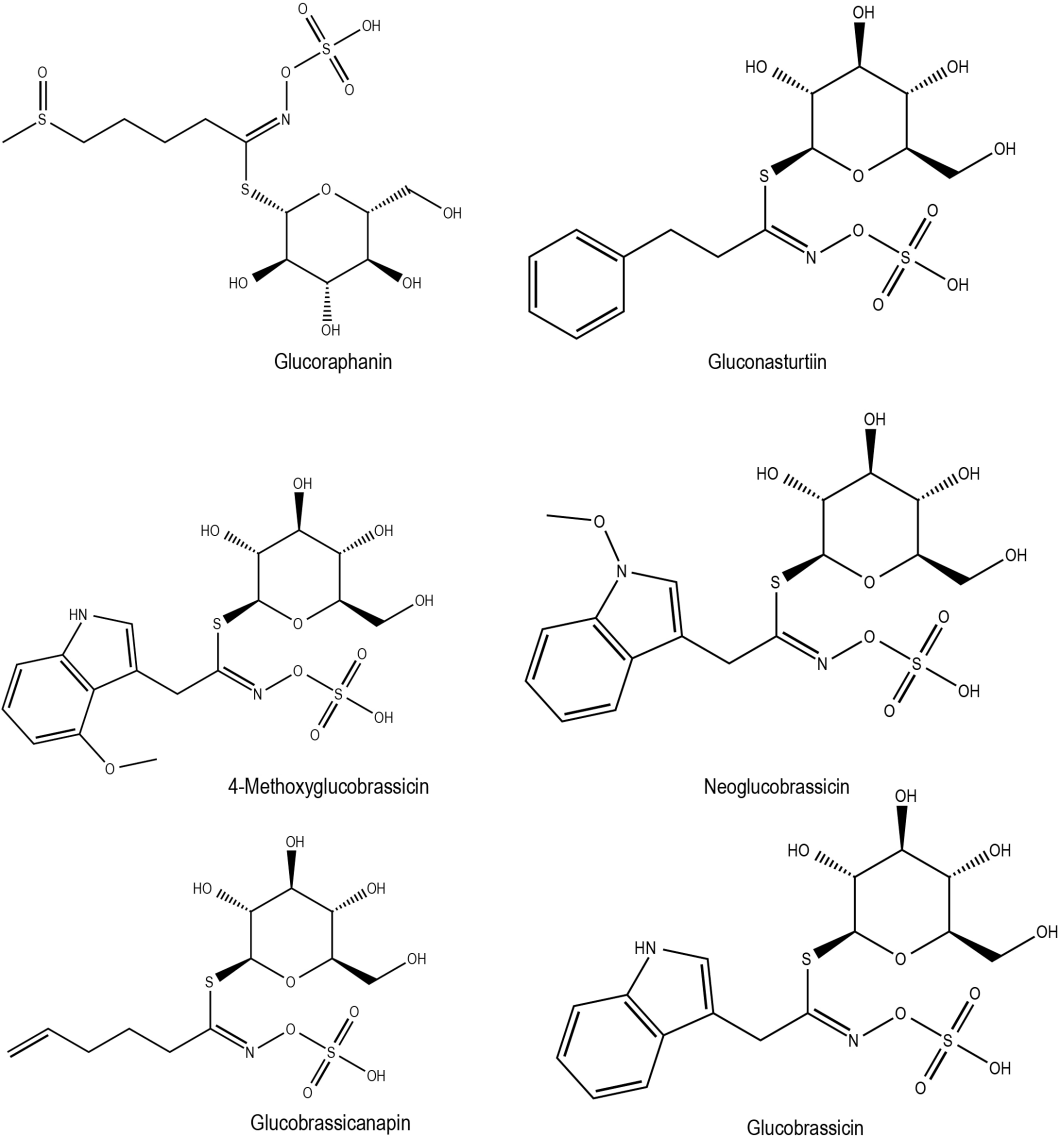
Chemical structures of glucosinolates with potential role in Brassica abiotic stress responses.

In the case of salinity stress (40-100 mM NaCl) in broccoli (*B. oleracea* var. *italica*), there was a reduction in the amount of individual and total aliphatic GSLs, as well as indolic GSLs, compared to controls (Sarikamiş and Çakir, 2017), possibly indicating GSLs break down upon exposure to stress. It is worth noting that GSL biosynthesis-related genes in *B. rapa* such as *BrMYBs* (*BrMYB28.1*, *BrMYB28.2*, *BrMYB34.3*, *BrMYB51.2, BrMYB28.1* and *BrMYB29.1*), were found to be differentially expressed based on the duration of stress exposure, as well as to different treatments like cold, drought, salt and ABA (Seo et al., 2017). This indicates the existence of complex regulatory system for GSL production in stress responses. The time dependent expression trend for GSL biosynthesis-related genes was also observed in the cold-stressed leaves of *B. juncea* (Tang et al., 2023a). While the contribution of time-dependent GSL production in cold stress warrants further study, it is clear that exogenous application of GSLs during cold stress confers cold-stress tolerance in *B. juncea* (Tang et al., 2023a). Interestingly, GSLs and ITCs (GSL derivatives) are also attributable to tastes such as umami, sourness, acidic bitterness, irritant and saltiness, bitterness, astringency, and richness. In some cases, stress-induced metabolic changes can negatively impact the crop nutritional attributes of crops. For instance, a high glucosinolate (GSL) concentration in edible oil seeds, such as rapeseed, can result in severe diseases of the liver, thyroid, and kidney if consumed (Wang et al., 2022). However, studies show that control over GSL accumulation is possible through efficient N management and variety selection to the meet the consumer demands (Wang et al., 2022). Chemical structures of some of the flavonoids with potential roles in abiotic stress responses in Brassica crops has been shown in **Figure 2**. Additionally, the GSL profiles and their potential stress response in Brassica crops published elsewhere listed in **Supplementary Table S1**.

## 3 Phenolic compounds

Phenolic compounds are ubiquitously present in plants and are found in variety of biological processes including plant stress responses. By evolution, plants had accumulated an array of phenolic compounds to adapt in changing environments (Kumar et al., 2023) (**Figure 1**). Phenolic compounds can be classified into two major groups flavonoids (e.g. anthocyanidins, flavanones, isoflavones and others) and non-flavonoids (e.g. Phenolic acids, lignins/lignans, coumarins, quinones, tannins, stilbenes, and others) based on the number and arrangement of the carbon atoms in their chemical structures (Cartea et al., 2011). Among phenolic compounds, flavonoids, phenolic acids, lignins/lignans, coumarins, quinones, tannins, stilbenes, cutin, suberin were either directly or indirectly known to participate in plant stress responses(Šamec et al., 2021a). Flavonoids and phenolic acids are two phenolic compounds were widely studied for their role in stress mitigation and also for their beneficial role in promoting human health.

### 3.1 Flavonoids

The flavonoid biosynthetic pathway is one of the most intensively investigated pathways for production of agronomically robust, stress-tolerant plants and natural dietary antioxidants (Ahmed et al., 2014). With more than 8000 flavonoids are found in plants, mostly with C6-C3-C6 structural framework, including chalcones, flavans, flavones, flavonols, anthocyanins, proanthocyanidins, with stilbenes being the only exception with C6-C2-C6 framework, synthesized through the flavonoid biosynthetic pathway (reviewed) (Chen et al., 2023b). Like other plants, flavonoids of Brassica mostly act as antioxidants thus helping the plants to overcome the adverse effects of major abiotic stresses such as drought, salinity, low and high temperature etc. A study detailing the zinc supplementation based salt tolerance mechanism in mustard [*Brassica juncea* (L.) Czern & Coss] has identified increase in flavonoid content contribute to stress tolerance (Ahmad et al., 2017) (**Supplementary Table S1**). Similarly, a mild drought stress in canola cultivars increased the flavonol and total flavonoid content (Rezayian et al., 2018). However, anthocyanin content showed a significant decrease in both cultivars under similar conditions. A study comparing yellow-coated and black-coated rape seeds of turnip rape (*Brassica rapa* L.) showed that a higher flavonoid content in black-seeded rapes was positively correlated with tolerance to NaCl-induced salinity stress (Xuan et al., 2015). High salinity induced the expression of Arabidopsis dihydroflavonol-4-reductase (DFR) in *B. napus*, increases the antioxidant efficiencies, possibly through anthocyanin accumulation (Kim et al., 2017). A study to investigate the exogenous application of liquiritoside on Chinese flowering cabbage (*Brassica rapa* subsp. *parachinensis*) for its impact on oxidative stress showed that enhanced accumulation of flavonoids, along with other phytochemicals, is helpful in mitigating the adverse effects (Akram et al., 2021). Apart from the major abiotic stress constraints, a recent study dealing with specific sound waves in phytochemical content of broccoli (*Brassica oleracea*) showed that a specific sound waves can significantly improve the flavonoid content (Kim et al., 2020). Flavonoids and their derivatives (flavonols and anthocyanins), as well as hydroxycinnamic acids, are the most common chelators of metal ions in *Brassica* plants, with the help of functional hydroxyl groups, in plants exposed to biotic and abiotic stresses, in addition to their radical scavenging activities (Salami et al., 2023). Low temperatures (8°C) increase the phytochemical (phenolic acids 3%; flavonoids 5%; carotenoids 15%; glucosinolates 21%) content in Kale (*Brassica oleracea* var. *acephala*) (Ljubej et al., 2021a). In contrast, short freezing (at −8°C, for 1 h after previous acclimation at 8°C, for 23 h) significantly reduced flavonoid content by 21% in the same crop. In another study, it was revealed that the total concentration of flavonoids remained unchanged despite declining temperature and global radiation in most of the kale cultivars under study. However, quercetin concentration increased while kaempferol decreased (Schmidt et al., 2010). Interestingly, the quercetin-to-kaempferol ratio was shown to increase in all of the investigated cultivars in response to decreasing temperature. Low temperature in *Brassica oleracea* var. *sabellica* enhance specific flavonol glycosides and hydroxycinnamic acid derivatives (Neugart et al., 2013; Rezayian and Zarinkamar, 2023). Similarly, exposure to cold and freezing temperatures in *Brassica napus* L. var. *oleifera* L. cv. Jantar significantly increases anthocyanin content (Solecka et al., 1999).

**Figure 3:**
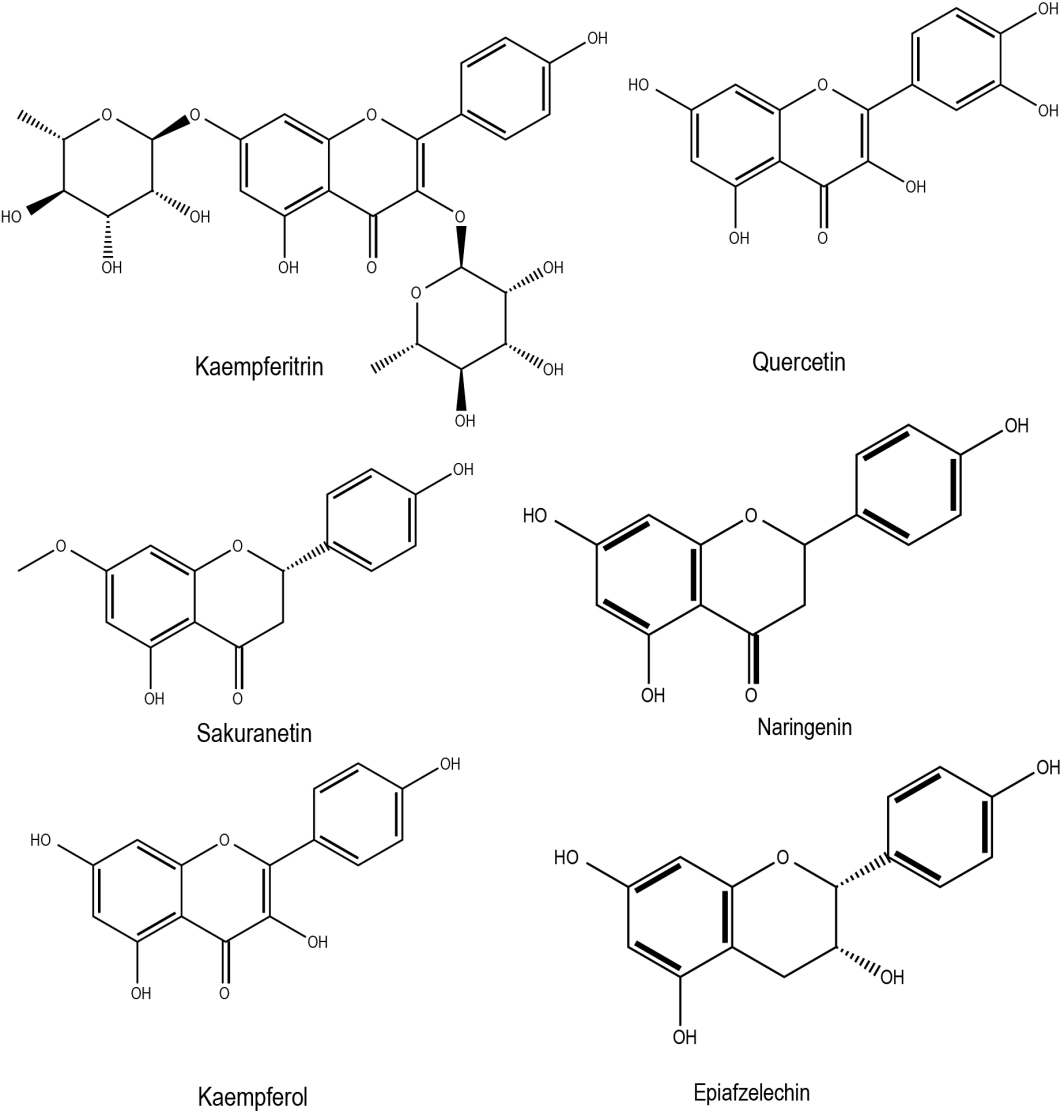
Chemical structures of flavonoids with potential role in Brassica abiotic stress responses.

In rapeseed, drought stress treatments with different moisture regimes increased phenolic compounds and flavonoids (Salami et al., 2023). Among other environmental factors, light and cold stress significantly enhanced anthocyanin accumulation (Loreti et al., 2008). Supplementation of zinc and calcium in salt stressed *Brassica juncea* was shown to mitigate salt stress adverse effects by enhancing antioxidant properties through several routes, including flavonoid accumulation (an increase of 86.19% over the control) (Ahmad et al., 2018). Selenium (Se) in plants has been shown to counteract major abiotic stresses such as cold, drought, water, salinity, and heavy metals by modulating the antioxidant system and various other unidentified pathways (Feng et al., 2013). Later, it was found that selenium induces flavonoid content to improve antioxidant capacities in turnip (*Brassica rapa* ssp. *rapa*) (Thiruvengadam and Chung, 2015). In general, abiotic stress conditions can indirectly regulate genes associated with flavonoid biosynthesis by modulating ABA homeostasis (Gai et al., 2020). It has been demonstrated that elevated ABA levels induce the production of flavones, anthocyanins, flavonols, isoflavones (e.g. kaempferitrin, sakuranetin, kaempferol) under drought conditions, which are beneficial in mitigating stress-induced damages (Loreti et al., 2008). It has been observed that an improved flavonoid content is beneficial in waterlogging stress conditions, as recently demonstrated in rape seed plants (Hong et al., 2023). This study also highlights the significant induction of flavonoids such as naringenin and epiafzelechin. Ahmed et al. (Ahmed et al., 2014) identified the association of dihydroflavonol 4-reductase (DFR)genes with anthocyanins and suggested that these genes could play a role in cold and freezing stress tolerance of *Brassica rapa*. Chemical structures of some of the flavonoids with potential roles in abiotic stress responses in Brassica crops has been shown in **Figure 3**. Studies have also shown that stress factor, Ultraviolet-B (UV-B) light, can induce the accumulation of flavonoids in Pakchoi (*Brassica rapa* L.) under specific light quantities (Hao et al., 2022). Almost all of the individual flavonoids identified in response to UV-B were relatively enhanced in seedlings treated with 4 µmol·m^−2^·s^−1^ UV-B for 4 h (Hao et al., 2022). Two flavonoids, kaempferol and quercetin appeared during anther development in *Brassica napus* has been shown to protect the haploidic pollen from UV light damage and water losses (Hsieh and Huang, 2007). The flavonoids found in Brassica crops can be useful in preventing cardiovascular diseases and some types of cancer, mainly by inhibiting LDL and DNA oxidation through scavenging peroxyl and hydroxyl radicals (Cao et al., 1997). The most important flavonoids in the human diet are quercetin and kaempferol, which are present as complex glycosides in Brassica species (Neugart and Bumke-Vogt, 2021).

### 3.2 Phenolic acids

Among the phenolic acids in Brassica vegetables, caffeic acid, *p*-coumaric acid, and ferulic acid have been reported in cabbages, while sinapic acid derivative were mainly found in Chinese cabbage and broccoli apart from cabbage (Park et al., 2014). The phenolic acid profiles of salt-tolerant Brassicas, such as kale and white cabbage, contained significantly higher level of total phenolic acids, including total hydroxycinnamic acids, when compared to salt-sensitive Chinese cabbage (Linić et al., 2019) (**Supplementary Table S1**). In contrast, hydroxybenzoic acids tended to decrease under the applied salinity conditions. Additionally, cell wall-bound phenolic acids, particularly sinapic acid (SiA), were found to be relatively higher in tolerant cultivars. In the case of salt-sensitive cultivars, a notable decrease was reported for protocatechuic acid (PA), 4-coumaric acid (pCoA), salicylic acid (SA), and caffeic acid (CaA) under salinity stress conditions. In fact, the offspring derived from salt-treated parental lines showed significantly higher phenolic acid contents and increased antioxidant capacities (Benincasa et al., 2021). In an attempt to use natural metabolites exogenously to ameliorate the adverse effects of high salinity stress in Chinese cabbage, foliar application of salicylic acid (SA) and ferulic acid (FA) (10–100 μM) had attenuating effects on salt-stressed plants (Linić et al., 2021). Further, it elevated the levels of phenolic acids, including ferulic acid and sinapic acid, endogenously along with flavonoids (KAE, and QUE). However, the endogenous SA levels were reduced in phenolic acid-treated plants growing under salt stress conditions. Another study demonstrated that the application of SA could ameliorate mild salinity stress effects in Indian mustard (*Brassica juncea* (L.) Czern. et Coss.) plants (Yusuf et al., 2008). In *B. napus*, SA application conferred salt stress tolerance by enhancing the activities of antioxidant defense and glyoxalase enzymes (Hasanuzzaman et al., 2014). The supplementation of SA (0.5 mM) in conjunction with 2.0 mM SO_4_^2−^ significantly improved maximum chlorophyll content, PS II efficiency, and gas exchange parameters compared to individual applications of 0.5 mM SA or 2.0 mM SO_4_^2−^ in salt- stressed mustard (*Brassica juncea* L.) (Rasheed et al., 2022). The study also explained the potential for SA to inhibit stress ethylene, while in combination with S, it may promote S-assimilation, thereby enhancing optimal ethylene formation and ethylene sensitivity to ultimately reverse salt stress. Moreover, seed priming with SA was shown to enhance the germination and growth responses of *B. napus* under drought and salinity stresses (Raees et al., 2022). Similarly, SA application detoxified the stress effects of NiCl_2_ and NaCl in *B. juncea*, improving growth, leaf water potential, pigment levels, and photosynthetic attributes (Yusuf et al., 2012). A recent study aiming to investigate the role of phenolic acids in salt tolerance of Chinese cabbage (*Brassica rapa* ssp*. pekinensis*), white cabbage (*Brassica oleracea* var. *capitata*), and kale (*Brassica oleracea* var. *acephala*) found that higher levels of hydroxycinnamic acids were positively correlated with salt tolerance in kale and white cabbage (Linić et al., 2019). In the case of salt-sensitive Chinese cabbage, the levels of caffeic, salicylic, and 4-coumaric acids were reduced.

The investigation of phenolic acids in drought-stressed Chinese cabbage (*Brassica rapa*) revealed that concentration of individual phenolic acids changes depending on the duration of the stress. After one week of drought stress, ferulic acid content was reduced compared to that of controls, while the amount of total phenolic acids increased over the controls (Shawon et al., 2020). The significance of salicylic acid (SA), also known as 2-hydroxybenzoic acid, as a signal molecule in inducing the plant defense responses, including local and systemic acquired resistance (SAR), is well-established in model plants (Simoh et al., 2010). In *B. napus*, pretreatment with salicylic acid alleviated drought-induced stress symptoms (La et al., 2019a). Furthermore, SA-mediated modulation coincided with the antagonistic downregulation of ABA-signaling genes. Additionally, SA pretreatment proved valuable in radical scavenging and enhanced proline synthesis, which likely helps plant maintains redox homeostasis. Similarly, Chinese cabbage pretreated with SA exhibited superior growth parameters and increased tolerance to drought stress conditions compared to the control group (La et al., 2019b). The exogenous application of nitric oxide (NO) in salt-stressed canola (*Brassica napus*) plants enhanced phenolic acids such as gallic acid and coumaric acid, along with other non-enzymatic antioxidants like catechin, diadzein, and kaempferol, in conjunction with antioxidant enzymes, thus promoting salt stress tolerance (Rezayian and Zarinkamar, 2023).

**Figure 4:**
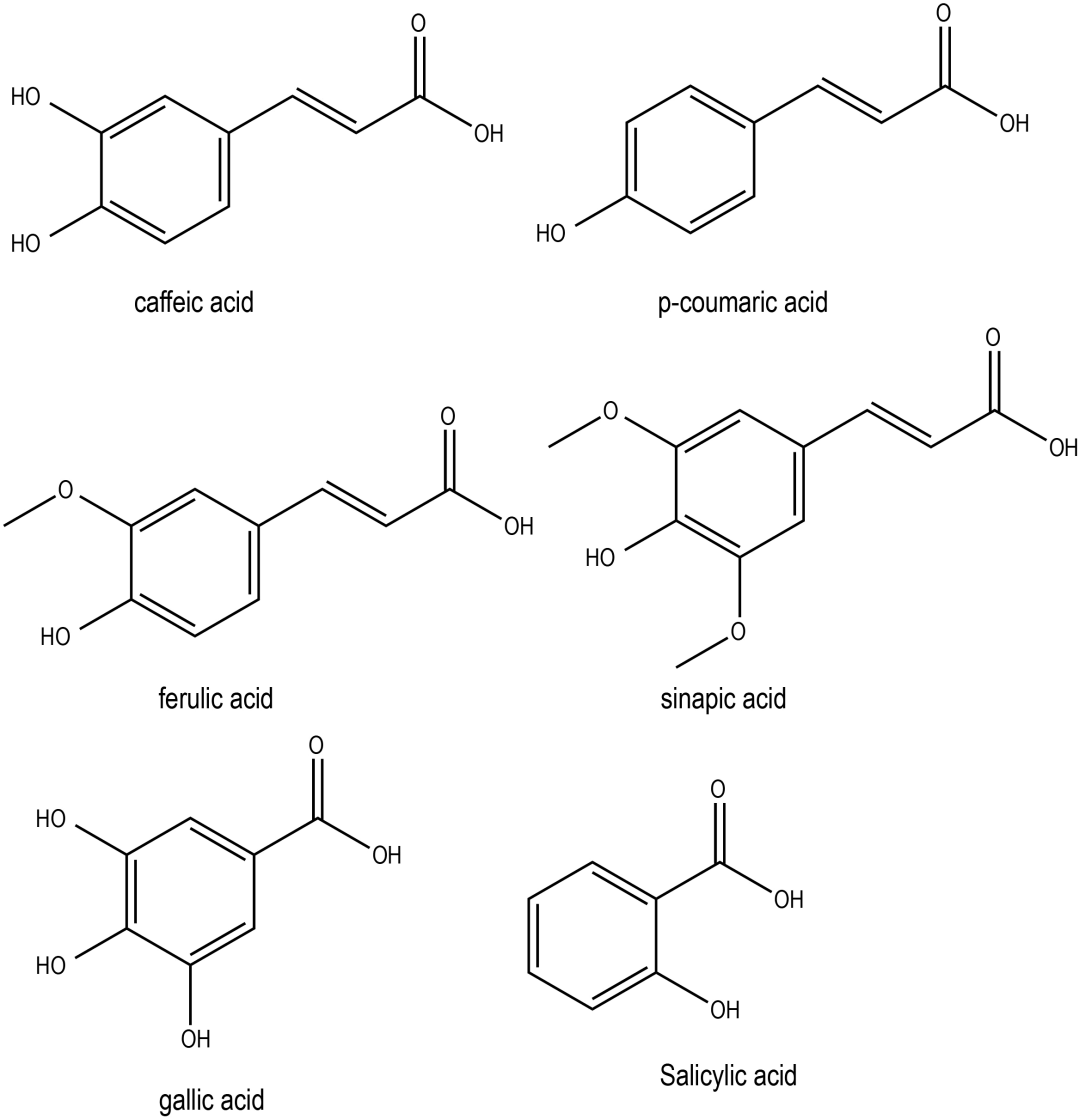
Chemical structures of phenolic acids with potential role in Brassica abiotic stress responses.

The levels of soluble *p*-coumaric, sinapic and ferulic acids increased approximately 3-, 4- and 5-fold, respectively, in winter oilseed rape leaves (*Brassica napus* L. var. *oleifera* L. cv. Jantar) exposed to cold and then to freezing treatments (Solecka et al., 1999). However, caffeic acid did not change during cold treatment. Cold-stressed heading-type kimchi cabbage (*B. rapa* L. ssp. *pekinensis*) accumulated phenolic acids, including p-coumaric, ferulic, and sinapic acids, which subsequently improved free-radical scavenging activity and antioxidant activity (Eom et al., 2022). Additionally, there were reports of phenolic acids improving the disease resistance in Brassica crops. For instance, pretreatment of *Brassica napus* plants with *p*-coumaric acid conferred resistance to black rot disease by inducing jasmonic acid-mediated phenolic accumulation (Islam et al., 2019). Exposure to cold and freezing temperatures in *Brassica napus* L. var. *oleifera* L. cv. Jantar significantly increased the soluble phenolic acid (e.g. p-coumaric, sinapic and ferulic acids) contents (Solecka et al., 1999). Conversely, high temperatures in winter crop *Brassica oleracea* var. *sabellica* favored the production of hydroxycinnamic acid derivatives such as disinapoyl-gentiobiose, while lower temperatures promoted the biosynthesis of sinapic acid-acylated flavonol tetraglycosides, such as kaempferol-3-O-sinapoyl-sophoroside-7-O-diglucoside (Neugart et al., 2016). Taken together, it is clear that phenolic acids contribute to abiotic stress tolerance in Brassica plant by enhancing the antioxidant efficiencies. Chemical structures of some of the phenolic acids with potential roles in abiotic stress responses in Brassica crops has been shown in **Figure 4**.

### 3.3 Other phenolic compounds

Among the various phenolic compounds found in Brassica crops, the lignin content in relation to stress responses has been fairly studied. In fact, a decade ago, it was proven that water-deficit stress in *B. napus* accumulates lignin content in leaves (Lee et al., 2014). In 2013, a study stated the possible molecular association between the enhanced expression of dirigent family genes and elevated soluble lignin content, contributing to tolerance against water stress in *Brassica rapa* crops (Thamil Arasan et al., 2013). A recent study detailing triacontanol-mediated drought tolerance in *Brassica juncea* L. found that elevated lignin levels or lignification processes helps plants reduce the negative consequences of drought stress (Ahmad et al., 2021). Other studies have demonstrated a positive correlation between enhanced lignin and disease resistance in *B. napus* (Wang et al., 2023). On the contrary, moderate salinity stress in *B. napus* has been shown to reduce the expression of genes associated with lignin biosynthesis pathways. Although, it is not clear whether this expression pattern is truly reflected in lignin content, it was hypothesized that it is an attempt by plants to preferentially allocate the energy for primary metabolism, eventually enhanced biomass production (Chen et al., 2023a). It is important to note that salinity stress (i.e. 150 mmol L^−1^ sodium chloride) in hydroponically grown canola, *Brassica napus* L., plants resulted in elevated levels of lignin (Hashemi et al., 2010). This study also has shown that silicon supplementation reduced lignin content under stress, improving the stress tolerance, possibly indicating the negative role of lignin accumulation in salinity tolerance. Lignin also plays a role in cadmium (Cd) detoxification processes in *Brassica chinensis* L (Wang et al., 2020b). The study concluded that Cd compartmentalization in the secondary cell wall by coupling with lignin side chain region, is responsible for Cd detoxification related to cultivar-dependent Cd accumulation. While it is known that lignin deposition could increase the mechanical strength of plant cells under stress, the mode of action in plant abiotic stresses mitigation mechanisms is not clear. The biological significance of stilbenes, tanins, cutins and other phenolic compounds were relatively understudied or unavailable in Brassica crops. However, preliminary studies are emerging to dissect their role in abiotic stress responses of Brassica crops. For instance, a recent study has shown that severe drought stress in *Brassica napus* L. significantly accumulates cutins, which might help the plants to avoid transpirational water loss during stress conditions (Fang et al., 2022).

## 4 Carotenoids

Carotenoids are a group of isoprenoid metabolites that play critical roles as light harvesting pigments and structural components of photosystems in plants. In general carotenoids can be divided into two classes: unsaturated C40 hydrocarbons as carotenes and their oxygenated derivatives known as xanthophylls (Ortega-Hernández et al., 2021). Carotenoids and its derivatives are crucial for the biosynthesis of phytohormones such as abscisic acid (ABA) and strigolactones (SLs), thus acting as signaling molecules in response to environmental and developmental cues (Sun et al., 2022). Carotenoid oxygenase is a key enzyme involved in the metabolism of carotenoids, showing differential expression pattern in response to abiotic stress conditions like salt, drought, and cold in both *B. rapa* and *B. oleracea* plants (Kim et al., 2016). Carotenoids are important, as their oxidized derivatives produced by specific carotenoid cleavage dioxygenases serve as photosynthetic accessory pigments, colorants, apocarotenoids, and are crucial for plant growth and development (Kim et al., 2016). A study by Kumar et al. (Kumar et al., 2013) identified carotenoid content as one of the simple indices for screening and identifying temperature stress-tolerant genotypes of *Brassica juncea*. Another study dealing with Kale (*B. oleracea*) under cold treatments found that individual carotenoid content varied at 15°C/10°C (Hwang et al., 2017). They found that the lutein, α-carotene, and β-carotene contents were very low, while the zeaxanthin contents was very high, and the ratio of individual to the total carotenoid content of kale was recorded as 4553% for carotene and 210% for zeaxanthin. Very recently, a study in Chinese cabbage (*Brassica rapa subsp. Pekinensis*) has revealed that a potential heat stress-tolerant candidate gene identified as *BraA09g011460.3C* (HD-zip I subfamily) has a significantly lower expression level in high carotenoid cultivars, possibly indicating a negative correlation with carotenoid content regulation in Chinese cabbage plants (Yin et al., 2023). At low temperature (8°C) for the short term, carotenoid content was induced by 15%, while other phytochemicals like phenolic acids, flavonoids, and glucosinolates contents were higher by 3%, 5%, and 21%, respectively, in kale (*Brassica oleracea* var. *acephala*) exposed to low temperature over the control plants (Ljubej et al., 2021a) (**Supplementary Table S1**). However, carotenoid content along with chlorophylls was reduced in most cases (Ben Ammar et al., 2022), which is aligned with previous findings. In fact, carotenoids and chlorophylls are functionally related and share common components in their biosynthesis.

**Figure 5:**
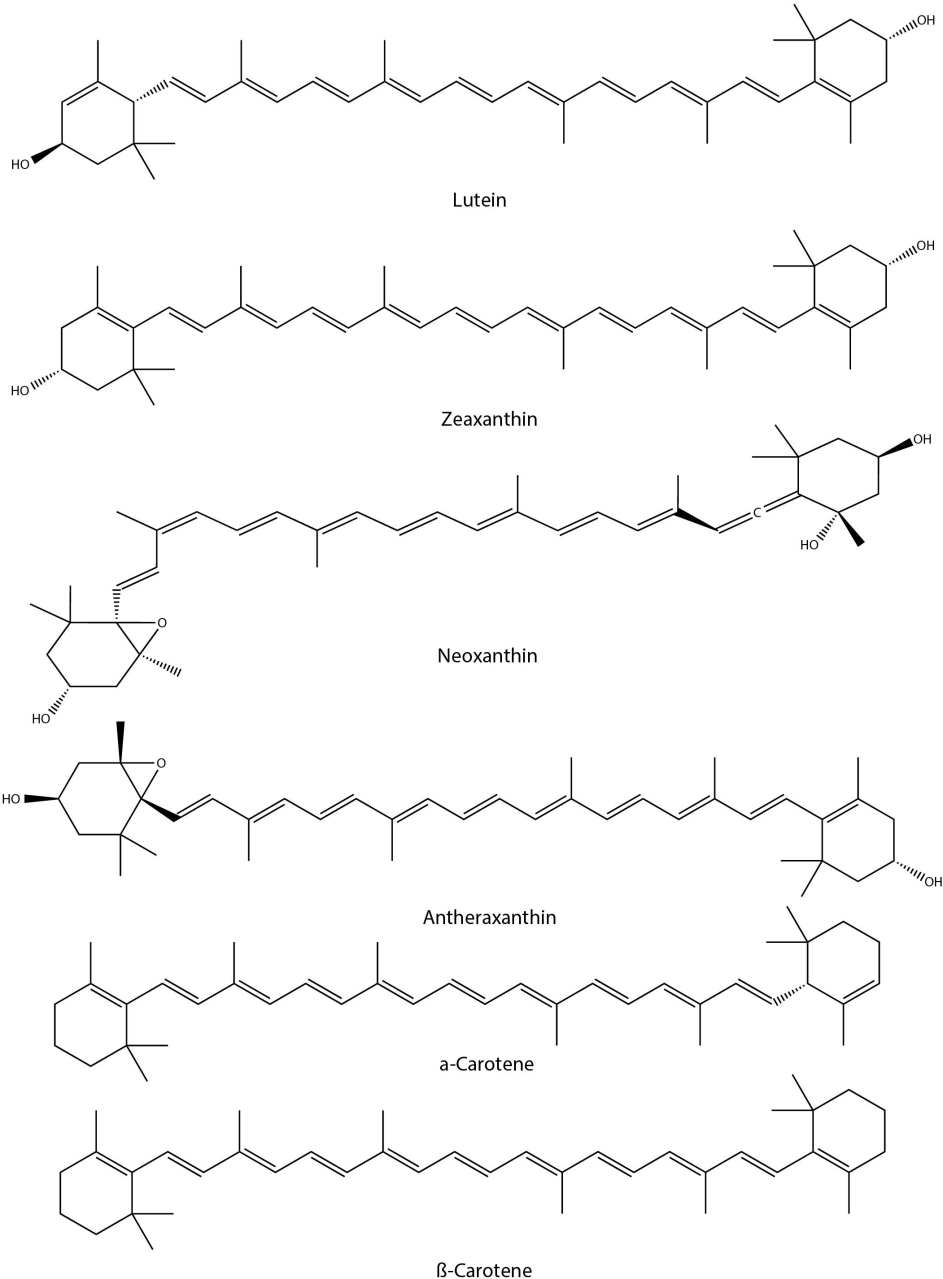
Chemical structures of carotenoids with potential role in Brassica abiotic stress responses.

Under drought conditions, the neoxanthin and antheraxanthin contents were significantly decreased in kale (*Brassica oleracea* L. var. *acephala*) (Barickman et al., 2020). Similarly, a significant reduction in total carotenoid content was reported for drought-stressed kale (*Brassica oleracea* var. *acephala*) leaves (Lee et al., 2017). The ratio of individual carotenoids to the total carotenoid content of kale leaves was reported as 51.4% for lutein, 4.44% for zeaxanthin, 2.76% for α-carotene, and 41.4% for β-carotene. UVB-treated (5 and 10 kJ m^−2^ d^−1^) canola (*Brassica napus* L.) seedlings were found to accumulate carotenoids and flavonoids (Sangtarash et al., 2009) compared to that of controls (0 kJ m^−2^ d^−1^). Whereas water stress reduced carotenoid content. Interestingly, water stress combined with enhanced UVB exposure also reduced carotenoid levels. Similarly, short-term waterlogging stress in kale (*Brassica oleracea* L. var. *acephala*) plants exhibited a decrease in carotenoid (CAR) concentrations of β-carotene, lutein, zeaxanthin, neoxanthin, and chlorophyll A (ChlA) and chlorophyll B (ChlB) concentrations (Brazel et al., 2021). In contrast, total glucosinolates content was found to be elevated. Nevertheless, the application of plant growth- promoting rhizobacteria (*Pseudomonas* sp. and *Staphylococcus* sp) and Biochar (*Morus alba* L. wood) in *Brassica napus* L. under drought conditions has positive growth impacts, found to be increased with carotenoid content along with other photosynthetic pigments (Lalay et al., 2022). In another study, SA pretreated Chinese cabbage plants retained carotenoid content during drought stress, which is similar to that of controls, while the non-treated drought-stressed plants showed a significant reduction (21.8%) in carotenoid content (La et al., 2019b). Application of 5-aminolevulinic acid (ALA) in rapeseed (*Brassica napus* L.) leaves under optimal growth conditions significantly increased carotenoid content (Liu et al., 2016). However, under drought stress conditions, similar treatment did not avert the drought impacts or failed to maintain carotenoid levels. This is despite the fact that carotenoids are involved in the stabilization and protection of the lipid phase of the thylakoid membrane and are also useful in scavenging radical species. *Brassica rapa ssp. rapa* seedlings exposed to environmentally hazardous copper oxide nanoparticles (CuO NPs) reduced the carotenoid, chlorophyll, and sugar content while increasing anthocyanins, glucosinolates and phenolic compounds. Reactive oxygen species (ROS), malondialdehyde (MDA), and hydrogen peroxide (H2O2) production were also enhanced in CuO NPs-treated seedlings (Chung et al., 2019). In another study, the Chinese Kale (*Brassica oleracea* var. *alboglabra*) sprouts under salinity stress conditions (160 mM Nacl solution) enhanced carotenoid content by 53% over the controls (Wang et al., 2020a). However, the carotenoid content does not positively correlate with the antioxidant capacities of the plants under study. On the other hand, salinity stress (50–200 mM NaCl) in Brassica leafy vegetables like Chinese cabbage, white cabbage, and kale did not significantly influence the carotenoid content (Šamec et al., 2021b). Among other factors, sulphur supplementation in kale sprouts does not cause any significant changes in carotenoid content (Kopsell et al., 2007). Similarly, the exogenous application of MeJA on kale plants does not exert any impact on carotenoid content (Sun et al., 2012). It is not clear whether enhanced carotenoid content can contribute stress tolerance. From the literature it is evident that abiotic stress conditions such as drought, waterlogging, temperature, and heavy metal stresses were reduced the total carotenoid content in Brassica crops while UVB exposure found to enhance the carotenoid content. To understand the biological significance further study detailing the functions of individual carotenoids in relation to stress responses are essential. Chemical structures of some of the carotenoids with potential roles in abiotic stress responses in Brassica crops has been shown in **Figure 5**.

## 5 Alkaloids

**Figure 6:**
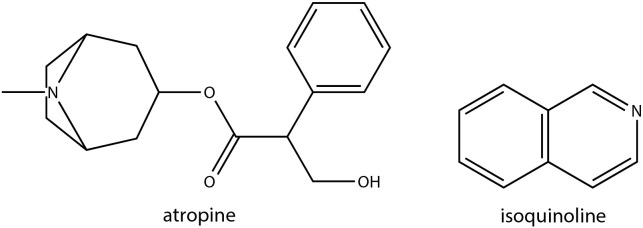
Chemical structures of alkaloids with potential role in Brassica abiotic stress responses.

Alkaloids are among the most abundant plant secondary metabolites, constituting approximately 20% of all plant-based secondary metabolites. They are predominantly solid compounds and possess antioxidant potential, which can be induced in response to both abiotic and biotic stress conditions (Huang et al., 2020; Tan et al., 2021). Alkaloids can be categorized into subgroups based on the basic heterocyclic nucleus in their structure which includes pyridine, tropane, isoquinoline, phenanthrene, phenylethylamine, indole, purine, imidazole and terpenoids (Pereira et al., 2023). Despite their biological significance in abiotic stress responses in other plants, alkaloids were relatively understudied in Brassica crops possibly due to their low availability in brassica vegetables. A study identified *Brassica napus* gene identified as Cab026133.1, encoding a tropinone reductase (BnTR1) was found to positively affect alkaloid metabolism, primarily producing atropine, ultimately enhancing low-temperature tolerance (LT) in BnTR1 overexpressing Arabidopsis plants (Huang et al., 2020) (**Supplementary Table S1**). Ectopically expressing BnTR1 in Arabidopsis plants significantly increased alkaloid content. In fact, the addition of 10 nmol of atropine per plant significantly rescued the susceptibility of control plants, thus confirming the role of alkaloids in LT tolerance. *B. juncea*, known for its excellent cadmium (Cd) accumulation and high phytoremediation potential, as well as its high biomass production in Cd-contaminated soil, has been shown to significantly accumulate alkaloids to counter the stress effects (Tan et al., 2021). An integrated transcriptomic and metabolomic analysis aimed at characterizing alkali stress responses in canola (*Brassica napus* L.) revealed that alkali stress in canola roots for 72 h differentially regulate isoquinoline alkaloid biosynthesis, among many other metabolic pathways (Wang et al., 2021). Similarly, an attempt to dissect the molecular basis of Cd stress response mechanisms in the hairy roots of *Brassica campestris* L. demonstrated that genes related isoquinoline alkaloid biosynthesis-related pathway were highly enriched under stress (Sun et al., 2023). Chemical structures of some of the alkaloids with potential roles in abiotic stress responses in Brassica crops has been shown in **Figure 6**.

## 6 Conclusions and future directions

**Figure 7:**
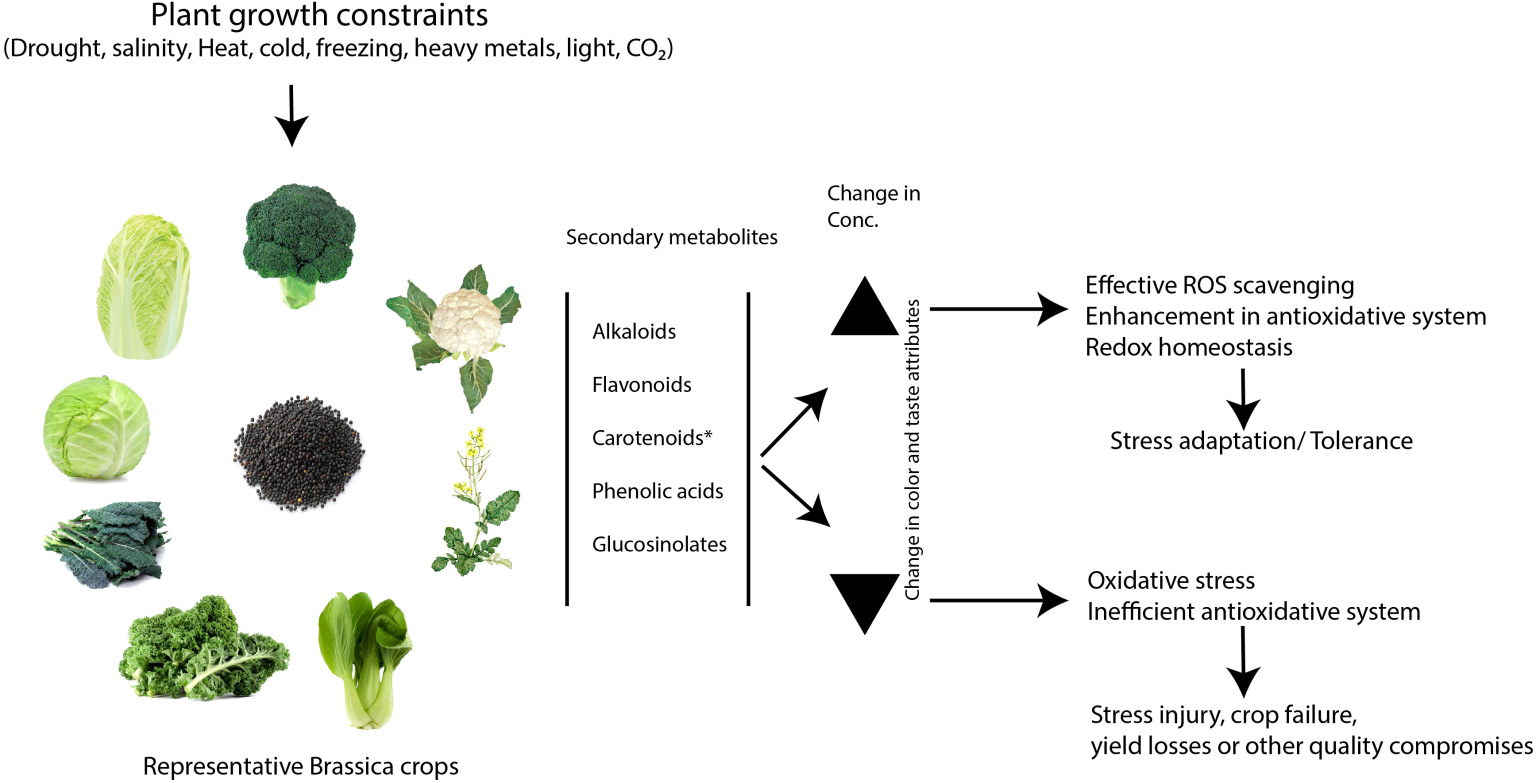
Metabolic adjustment of secondary metabolite biosynthesis and its implications in plant stress responses. The figure represents the hypothetical model illustrating the potential roles of secondary metabolites, such as glucosinolates, flavonoids, carotenoids, phenolic acids, and alkaloids, during stress-adaptive metabolic changes in Brassica crops, based on the literature. In figure, * represents the lack of evidence supporting the positive correlation between carotenoid content and stress tolerance in Brassica against major abiotic stresses.

Major abiotic stresses differentially regulate the content of secondary metabolites, such as glucosinolates, carotenoids, flavonoids, phenolic acids, and alkaloids in Brassica crops. In general, changes in the content of these metabolites have implications for the plant’s stress responses. In some cases, they act as stress mitigators by functioning as non-enzymatic antioxidants. Therefore, their elevated levels are expected to help the plants avert the negative consequences of adverse stress conditions (**Figure 7**). Nevertheless, specific compounds within the classes have shown differential changes in content under similar environmental conditions. This necessitates further study of individual compounds to decipher their biological significance in plant metabolic adaptation under adverse growth conditions. While stress-induced metabolites serve multiple functions in plants, they are mostly known for their role in countering oxidative stress resulting from the excessive accumulation of reactive oxygen species during stress. It is important to note that plants possess vast arrays of defense chemicals and are responsive to environmental conditions. Any variation in the content of these metabolites under stress may reveal stress-responsive traits. However, in most model and non-model plants, the study of plant stress metabolic adaptation has been limited to few classes of secondary metabolites, such as glucosinolates, flavonoids, carotenoids, alkaloids and phenolic acids. This indicates that a larger fraction of secondary metabolites and their biological significance in plant stress response is yet to be identified. Furthermore, several known and unknown factors found to be synchronized with most of the abiotic stress conditions, altering the secondary metabolite production. This adds to the challenges in precisely deciphering the particular metabolite production pathway in stress adaptation. Detailed study of the genetic basis of secondary metabolite production pathway is needed to generate the resources that can facilitate metabolic engineering to achieve stress-resilient crop production. However, most of the key metabolite production pathways are still emerging or limited in their harnessing of nutraceutical importance for human and animal diets, rather than addressing stress mitigation mechanisms. The application of next-generation metabolomics using high-throughput MS techniques, coupled with robust bioinformatics data analysis platform, is expected to generate precise and accurate metabolite datasets for specific stress conditions. It is interesting to note that identification of effective stress-mitigating metabolites or their synthetic decoys can accelerate crop stress management programs through exogenous applications. This study clearly shows that the identification of stress-response metabolites will enhance our understanding of complex networks and interplay of associated systems in plants during abiotic stresses, which is more useful for comprehending plant stress adaptability.

## Author contributions

MM: Conceptualization, Data curation, Formal analysis, Writing – original draft, Writing – review & editing. SL: Conceptualization, Data curation, Formal analysis, Funding acquisition, Project administration, Resources, Writing – review & editing.
